# 

**Published:** 1893-05

**Authors:** 


Conference of State Medical Examining and Licensing Boards.—The third annual meeting of the Conference of State Medical Examining and Licensing Boards will be held in Milwaukee, Wis., June 7, 1893. The following subjects will be discussed, and we will be glad to have you notify us of your intention to prepare a paper on some one of them, and take part in the discussion of any or all of them. We will also be glad to have you suggest, in advance of the meeting, other subjects you may think it desirable to discuss.

I. The Evolution of State Medical Examining and Licensing Boards. Their present and prospective influence in elevating the moral and intellectual tone of the profession.

II. Composition of Boards. (*a*) The desirable number of members. (*b*) The desirable appointing power. (*c*) The advantages and disadvantages of separate boards representing the different schools of practice.

III. Provisions of the Various State Laws. (*a*) Should the possession of a diploma from a recognized medical school be a prerequisite to appearing before a board for examination? (*b*) What reciprocal relations should exist between boards? (*c*) Should teachers in medical schools be eligible to membership on State Examining Boards? (*d*) Defects in existing laws, the best law in vogue, the ideal law.

IV. Methods of Conducting Examinations. (*a*) How should the examination be prepared? (*b*) The scope of examinations. (*c*) The minimum and maximum requirements.

HUGH M. TAYLOR, M. D., *Sec'y and Treas.* JOHN H. RAUCH, M. D., *President.*

---

ELEVENTH INTERNATIONAL CONGRESS.—Immediately following the session of the coming Pan-American Medical Congress, a special line of ocean steamships will be at the service of the delegates and all others who desire to visit the Eternal City on the occasion of the Eleventh International Medical Congress, the sessions of which will begin September 24th and continue till October 1st.

The admission fee for the International Congress is placed at the nominal sum of $5; and Dr. A. Jacobi, Chairman of the American National Committee, has volunteered to forward applications and fee for all who may desire him to do so; otherwise application should be made to the International Treasurer, Prof. L. Pagliani, Rome, Italy.

Papers intended for the Congress must be announced on or before June 30th, and a brief abstract of the same, with conclusions, sent to the committee on or before July 31st.

Italian, French, English, and German will be the official languages.

Reduced rates of fare, and facilities for visiting noted localities in Italy, have been provided.

---

AMERICAN ELECTRO-THERAPEUTIC ASSOCIATION.—At the second annual meeting of the American Electro-Therapeutic Association the following officers were elected for the ensuing year:

President, Dr. Augustin H. Goelet, 531 West Fifty-seventh street, New York; First Vice-President, Dr. Wm. F. Hutchinson, Providence, R. I.; Second Vice-President, Dr. W. J. Herdman, Ann Arbor, Mich.; Secretary, Dr. Margaret A. Cleaves, 68 Madison avenue, New York; Treasurer, R. J. Nunn, 119 York street, Savannah, Ga.

The third annual meeting will be held in Chicago on September 12, 13, and 14, 1893. A cordial invitation is extended to all members of the profession interested in electro-therapeutics. Arrangements for special rates on railways and at hotels are in progress.

The Committee of Arrangements will be obliged if those who intend being present at the meeting will send their names, the class and amount of accommodation required, titles of papers to be presented, applications for membership, etc., at as early a date as possible. Accommodations should be secured early, on account of the crowded condition of the hotels, because of the World's Fair. All communications should be addressed to the secretary.

The committee will be glad to furnish any information in regard to the meeting, upon application.

The Committee of Arrangements,

FRANKLIN H. MARTIN, *Chairman.* S. C. STANTON, *Secretary.*

### BOOKS RECEIVED.

The Diseases of the Nervous System. A Text-book for Physicians and Students. By Dr. Ludwig Hirt, Professor at the University of Breslau. Translated, with permission of the author, by August Hoch, M. D., assisted by Frank R. Smith, A. M., (Cantab.,) M. D., Assistant Physician to the Johns Hopkins Hospital. With an introduction by William Osler, M. D., F. R. C. P., Professor of Medicine in the Johns Hopkins University, etc. With 178 illustrations. Large octavo, pp. xv.—683. New York: D. Appleton & Co. 1893.

An Introduction to the Study of the Diseases of the Skin. By P. H. Pye-Smith, M. D., F. R. S., F. R. C. P., Physician to the Department of Cutaneous Diseases in Guy's Hospital, London. In one handsome 12mo volume of 407 pages, with twenty-eight illustrations, eighteen of which are colored. Cloth, $2.00. Philadelphia: Lea Brothers & Co. 1893.

Elementary Physiology for Students. By Alfred T. Schofield, M. D., M. R. C. S., Late House Physician to the London Hospital; Special Lecturer, National Health Society. In one handsome 12mo volume of 385 pages, with 227 engravings and two colored plates. Cloth, $2.00. Philadelphia: Lea Brothers & Co.

Modern Gynecology: A Treatise on Diseases of Women, comprising the results of the latest investigations and treatment in this branch of medical science. By Charles H. Bushong, M. D., Assistant Gynecologist to the Demilt Dispensary, New York; formerly Attending Physician to the Northern Dispensary, and Assistant to the Vanderbilt Clinic, College of Physicians and Surgeons, New York. Illustrated. Octavo volume, pp. 380. Price, $2.75. New York: E. B. Treat, 5 Cooper Union. 1893.

